# Supplementary material for: The rhizosphere and root selections intensify fungi-bacteria interaction in abiotic stress-resistant plants
Source: PeerJ. 2024 Apr 15;12:e17225. doi: 10.7717/peerj.17225 (PMC11025542; doi:10.7717/peerj.17225)

Table S1 The sample and amplicon sequencing information in this study.

| Sample name | Host species | Host  abbreviation | Compartment | Fungal community | | | | Bacterial community | | | |
| --- | --- | --- | --- | --- | --- | --- | --- | --- | --- | --- | --- |
|  |  |  |  | Total reads | Clean reads | Unclassified reads | No. of ASVs | Total reads | Clean reads | Unclassified reads | No. of ASVs |
| B47 | Bulk soil | Bulk | Bulk | 51493 | 41886 | 8103 | 971 | 80123 | 71288 | 24 | 6749 |
| B59 | Bulk soil | Bulk | Bulk | 55697 | 46677 | 7471 | 1182 | 80692 | 69402 | 2 | 7189 |
| B57 | Bulk soil | Bulk | Bulk | 44287 | 42637 | 327 | 1264 | 79533 | 63576 | 0 | 7667 |
| B81 | Bulk soil | Bulk | Bulk | 79111 | 77556 | 142 | 1585 | 67594 | 55130 | 0 | 6297 |
| B67 | Bulk soil | Bulk | Bulk | 78506 | 77047 | 168 | 1276.9 | 67916 | 52398 | 0 | 6856 |
| B51 | Bulk soil | Bulk | Bulk | 57331 | 44798 | 10912 | 1041.9 | 74806 | 65612 | 1 | 6512 |
| B83 | Bulk soil | Bulk | Bulk | 76613 | 74936 | 444 | 1526.9 | 59373 | 52174 | 1 | 5565 |
| B73 | Bulk soil | Bulk | Bulk | 70201 | 68266 | 417 | 1858 | 54470 | 44560 | 0 | 5586 |
| B65 | Bulk soil | Bulk | Bulk | 55562 | 44514 | 9581 | 1081 | 75042 | 66388 | 1 | 7448 |
| B63 | Bulk soil | Bulk | Bulk | 81928 | 80377 | 168 | 1335 | 74050 | 61114 | 0 | 7646 |
| B53 | Bulk soil | Bulk | Bulk | 51081 | 43681 | 5514 | 1365 | 64267 | 60846 | 0 | 3887 |
| B49 | Bulk soil | Bulk | Bulk | 43999 | 42664 | 206 | 1860 | 67753 | 54493 | 0 | 6571 |
| B45 | Bulk soil | Bulk | Bulk | 75017 | 73674 | 173 | 1636.9 | 80625 | 67585 | 0 | 7738 |
| B41 | Bulk soil | Bulk | Bulk | 69851 | 68673 | 51 | 1491 | 73978 | 64481 | 0 | 7433 |
| B61 | Bulk soil | Bulk | Bulk | 84473 | 81695 | 735 | 1436 | 64955 | 48277 | 0 | 6584 |
| B71 | Bulk soil | Bulk | Bulk | 79328 | 77938 | 151 | 1760.7 | 78008 | 69396 | 3 | 7387 |
| B55 | Bulk soil | Bulk | Bulk | 75273 | 73895 | 177 | 1314 | 79578 | 61193 | 0 | 7026 |
| B66 | *Fagraea ceilanica* | FC | Rhizosphere | 84391 | 83124 | 48 | 1317.9 | 54614 | 45960 | 0 | 4913 |
| C33 | *Fagraea ceilanica* | FC | Rhizosphere | 83438 | 82440 | 54 | 987 | 74146 | 60083 | 0 | 5386 |
| C34 | *Fagraea ceilanica* | FC | Rhizosphere | 56001 | 54850 | 115 | 795.9 | 79042 | 67110 | 0 | 5263 |
| C68 | *Fagraea ceilanica* | FC | Rhizosphere | 86241 | 85021 | 64 | 1092 | 48533 | 39535 | 0 | 3672 |
| B72 | *Hernandia sonora* | HS | Rhizosphere | 57208 | 55753 | 57 | 1295 | 77970 | 64398 | 1 | 7045 |
| C39 | *Hernandia sonora* | HS | Rhizosphere | 77445 | 76493 | 171 | 885 | 84888 | 75481 | 1 | 3350 |
| C40 | *Hernandia sonora* | HS | Rhizosphere | 73700 | 71439 | 745 | 1426.8 | 66392 | 51847 | 0 | 4805 |
| C70 | *Hernandia sonora* | HS | Rhizosphere | 62811 | 57730 | 3455 | 1707.9 | 83779 | 74127 | 0 | 6417 |
| B62 | *Melaleuca bracteata* | MB | Rhizosphere | 87399 | 84617 | 274 | 1215 | 74667 | 55716 | 0 | 6788 |
| C27 | *Melaleuca bracteata* | MB | Rhizosphere | 84595 | 79126 | 3504 | 1350.8 | 56020 | 47558 | 0 | 4417 |
| C28 | *Melaleuca bracteata* | MB | Rhizosphere | 87228 | 85024 | 528 | 1019.9 | 73616 | 56860 | 0 | 5339 |
| C71 | *Melaleuca bracteata* | MB | Rhizosphere | 73163 | 72298 | 246 | 897 | 81289 | 60438 | 2 | 6553 |
| C47 | *Pongamia pinnata* | PP | Rhizosphere | 84606 | 80086 | 2476 | 713 | 50601 | 38735 | 1 | 4087 |
| C48 | *Pongamia pinnata* | PP | Rhizosphere | 75245 | 72712 | 1331 | 981 | 60791 | 50526 | 4 | 3474 |
| C67 | *Pongamia pinnata* | PP | Rhizosphere | 83827 | 64204 | 17326 | 749 | 85672 | 81531 | 5 | 4318 |
| B44 | *Portulaca pilosa* | PPI | Rhizosphere | 50611 | 44563 | 5017 | 1045 | 80499 | 66598 | 0 | 7596 |
| C11 | *Portulaca pilosa* | PPI | Rhizosphere | 63282 | 57752 | 4066 | 1492 | 72086 | 55364 | 0 | 4123 |
| C12 | *Portulaca pilosa* | PPI | Rhizosphere | 73809 | 67176 | 4510 | 1521 | 73941 | 59184 | 0 | 4072 |
| C75 | *Portulaca pilosa* | PPI | Rhizosphere | 75019 | 71138 | 1271 | 1735 | 51307 | 39298 | 0 | 3835 |
| B42 | *Ruellia brittoniana* | RB | Rhizosphere | 53317 | 42263 | 9697 | 978 | 53474 | 46164 | 0 | 6033 |
| C7 | *Ruellia brittoniana* | RB | Rhizosphere | 75311 | 74044 | 347 | 920 | 79932 | 77032 | 25 | 2427 |
| C69 | *Ruellia brittoniana* | RB | Rhizosphere | 85817 | 84450 | 305 | 960 | 88697 | 79223 | 0 | 6711 |
| B40 | *Scaevola sericea* | SS | Rhizosphere | 59276 | 57366 | 204 | 1939.9 | 84860 | 73336 | 1 | 7892 |
| C1 | *Scaevola sericea* | SS | Rhizosphere | 77993 | 73900 | 642 | 1219.9 | 73885 | 56305 | 0 | 4866 |
| C2 | *Scaevola sericea* | SS | Rhizosphere | 89993 | 85535 | 1636 | 1501 | 68783 | 55632 | 0 | 4618 |
| C73 | *Scaevola sericea* | SS | Rhizosphere | 82395 | 80573 | 149 | 1461 | 46660 | 32581 | 0 | 2792 |
| C64 | *Casuarina equisetifolia* | CE | Rhizosphere | 72666 | 70506 | 47 | 1048 | 46416 | 32417 | 0 | 4696 |
| C65 | *Casuarina equisetifolia* | CE | Rhizosphere | 61756 | 58889 | 1002 | 1048 | 81774 | 65445 | 0 | 5447 |
| C81 | *Casuarina equisetifolia* | CE | Rhizosphere | 87798 | 85526 | 164 | 1274 | 87065 | 69268 | 1 | 6064 |
| B74 | *Casuarina equisetifolia* | CE | Rhizosphere | 72076 | 71190 | 51 | 1134.9 | 85324 | 76239 | 0 | 7217 |
| C56 | *Calophyllum inophyllum* | CI | Rhizosphere | 83793 | 81548 | 527 | 1113.6 | 85550 | 65772 | 0 | 5947 |
| C57 | *Calophyllum inophyllum* | CI | Rhizosphere | 84161 | 80675 | 1227 | 1572.8 | 70658 | 50232 | 0 | 5908 |
| C77 | *Calophyllum inophyllum* | CI | Rhizosphere | 78123 | 76008 | 363 | 1477.9 | 81331 | 60589 | 0 | 5350 |
| B58 | *Calophyllum inophyllum* | CI | Rhizosphere | 81861 | 80973 | 41 | 1195.7 | 75266 | 63299 | 1 | 6615 |
| C60 | *Guettarda speciosa* | GS | Rhizosphere | 86042 | 84124 | 1023 | 911 | 54254 | 45650 | 26 | 4587 |
| C61 | *Guettarda speciosa* | GS | Rhizosphere | 57596 | 54143 | 1316 | 1688 | 81285 | 61955 | 0 | 6012 |
| C79 | *Guettarda speciosa* | GS | Rhizosphere | 75735 | 74406 | 288 | 1208.9 | 51619 | 43280 | 0 | 4862 |
| B50 | *Guettarda speciosa* | GS | Rhizosphere | 66233 | 63687 | 1264 | 1447 | 86221 | 75280 | 0 | 7373 |
| C58 | *Heritiera littoralis* | HL | Rhizosphere | 53411 | 50477 | 1500 | 1195 | 56733 | 52567 | 0 | 4431 |
| C59 | *Heritiera littoralis* | HL | Rhizosphere | 72061 | 68725 | 2400 | 1171 | 49069 | 44190 | 6 | 3591 |
| C78 | *Heritiera littoralis* | HL | Rhizosphere | 82890 | 78432 | 2485 | 1155.9 | 86183 | 60323 | 1 | 5000 |
| B82 | *Heritiera littoralis* | HL | Rhizosphere | 86910 | 85483 | 350 | 1382 | 73329 | 62374 | 0 | 5338 |
| C35 | *Fagraea ceilanica* | FC | Root | 77435 | 76573 | 385 | 398 | 68037 | 58795 | 4 | 3864 |
| C36 | *Fagraea ceilanica* | FC | Root | 60477 | 59716 | 316 | 462.9 | 78330 | 68339 | 0 | 4160 |
| C37 | *Fagraea ceilanica* | FC | Root | 80330 | 79564 | 74 | 572 | 86727 | 83956 | 1 | 2090 |
| C38 | *Fagraea ceilanica* | FC | Root | 82097 | 81280 | 163 | 504 | 76730 | 75068 | 0 | 1378 |
| C41 | *Hernandia sonora* | HS | Root | 82172 | 79688 | 915 | 621 | 60696 | 56238 | 3 | 2819 |
| C42 | *Hernandia sonora* | HS | Root | 75118 | 72852 | 587 | 617 | 67501 | 63823 | 3 | 1374 |
| C43 | *Hernandia sonora* | HS | Root | 78352 | 75210 | 1045 | 653 | 81489 | 80384 | 29 | 1213 |
| C29 | *Melaleuca bracteata* | MB | Root | 73985 | 50768 | 21325 | 483 | 83983 | 78455 | 0 | 3013 |
| C30 | *Melaleuca bracteata* | MB | Root | 49324 | 39598 | 8605 | 413 | 73492 | 68104 | 0 | 3298 |
| C31 | *Melaleuca bracteata* | MB | Root | 85569 | 61555 | 22447 | 912 | 82793 | 80513 | 1 | 1932 |
| C32 | *Melaleuca bracteata* | MB | Root | 60683 | 58897 | 1230 | 558 | 74620 | 72435 | 2 | 2071 |
| C49 | *Pongamia pinnata* | PP | Root | 70605 | 68673 | 100 | 921 | 86486 | 84278 | 0 | 2935 |
| C51 | *Pongamia pinnata* | PP | Root | 64740 | 61319 | 2256 | 970 | 51314 | 43339 | 0 | 4030 |
| C13 | *Portulaca pilosa* | PPI | Root | 80735 | 75414 | 2011 | 550.9 | 70492 | 68623 | 0 | 1511 |
| C14 | *Portulaca pilosa* | PPI | Root | 76889 | 72824 | 1438 | 411 | 67712 | 65625 | 0 | 1501 |
| C15 | *Portulaca pilosa* | PPI | Root | 66026 | 60979 | 2601 | 351 | 74325 | 71930 | 0 | 1631 |
| C16 | *Portulaca pilosa* | PPI | Root | 80872 | 77345 | 444 | 366 | 53053 | 51243 | 0 | 1269 |
| C8 | *Ruellia brittoniana* | RB | Root | 76627 | 75591 | 14 | 529 | 74705 | 70107 | 3 | 2648 |
| C9 | *Ruellia brittoniana* | RB | Root | 63346 | 62798 | 45 | 480 | 68105 | 58267 | 0 | 4314 |
| C10 | *Ruellia brittoniana* | RB | Root | 89820 | 88806 | 129 | 446.8 | 67895 | 61355 | 0 | 3166 |
| C3 | *Scaevola sericea* | SS | Root | 73943 | 73730 | 21 | 261 | 63512 | 57761 | 0 | 2780 |
| C4 | *Scaevola sericea* | SS | Root | 84576 | 84005 | 40 | 450 | 67658 | 60681 | 0 | 3855 |
| C5 | *Scaevola sericea* | SS | Root | 85559 | 85253 | 6 | 398 | 66819 | 61733 | 0 | 2362 |
| C44 | *Casuarina equisetifolia* | CE | Root | 89655 | 88794 | 182 | 628 | 55256 | 53501 | 1 | 2192 |
| C45 | *Casuarina equisetifolia* | CE | Root | 79092 | 78352 | 38 | 904 | 88978 | 85732 | 1 | 3495 |
| C46 | *Casuarina equisetifolia* | CE | Root | 77853 | 77384 | 53 | 565 | 78541 | 74733 | 0 | 3430 |
| C20 | *Calophyllum inophyllum* | CI | Root | 90828 | 89686 | 581 | 431 | 47368 | 45732 | 1 | 1763 |
| C21 | *Calophyllum inophyllum* | CI | Root | 92308 | 91585 | 284 | 405 | 78462 | 76501 | 2 | 1186 |
| C22 | *Calophyllum inophyllum* | CI | Root | 85026 | 84229 | 275 | 435.9 | 85222 | 83201 | 1 | 1367 |
| C23 | *Calophyllum inophyllum* | CI | Root | 90906 | 90055 | 650 | 198 | 66038 | 65471 | 0 | 660 |
| C24 | *Calophyllum inophyllum* | CI | Root | 76928 | 76414 | 56 | 260 | 85389 | 83344 | 4 | 1427 |
| C17 | *Guettarda speciosa* | GS | Root | 79649 | 78292 | 1096 | 256 | 78959 | 77421 | 0 | 1061 |
| C18 | *Guettarda speciosa* | GS | Root | 89560 | 88880 | 253 | 266 | 71641 | 68098 | 0 | 1274 |
| C19 | *Guettarda speciosa* | GS | Root | 89220 | 87924 | 815 | 298 | 67731 | 63972 | 0 | 1445 |
| C52 | *Heritiera littoralis* | HL | Root | 52312 | 51096 | 170 | 1065.9 | 61824 | 52712 | 0 | 3554 |
| C53 | *Heritiera littoralis* | HL | Root | 52006 | 50984 | 103 | 1023 | 76254 | 61816 | 0 | 5750 |
| C54 | *Heritiera littoralis* | HL | Root | 60666 | 59538 | 364 | 753 | 74068 | 62577 | 0 | 4901 |
| C55 | *Heritiera littoralis* | HL | Root | 39994 | 39127 | 140 | 916 | 74031 | 67799 | 0 | 3727 |

|  |
| --- |

|  |
| --- |

|  |
| --- |

Figure S1 The rarefaction curves of the sequenced fungal (A) and bacteria (B) communities in this study. The number of fungal ITS and bacteria 16S rRNA sequences were normalized at 39, 262 and 37, 161, respectively.


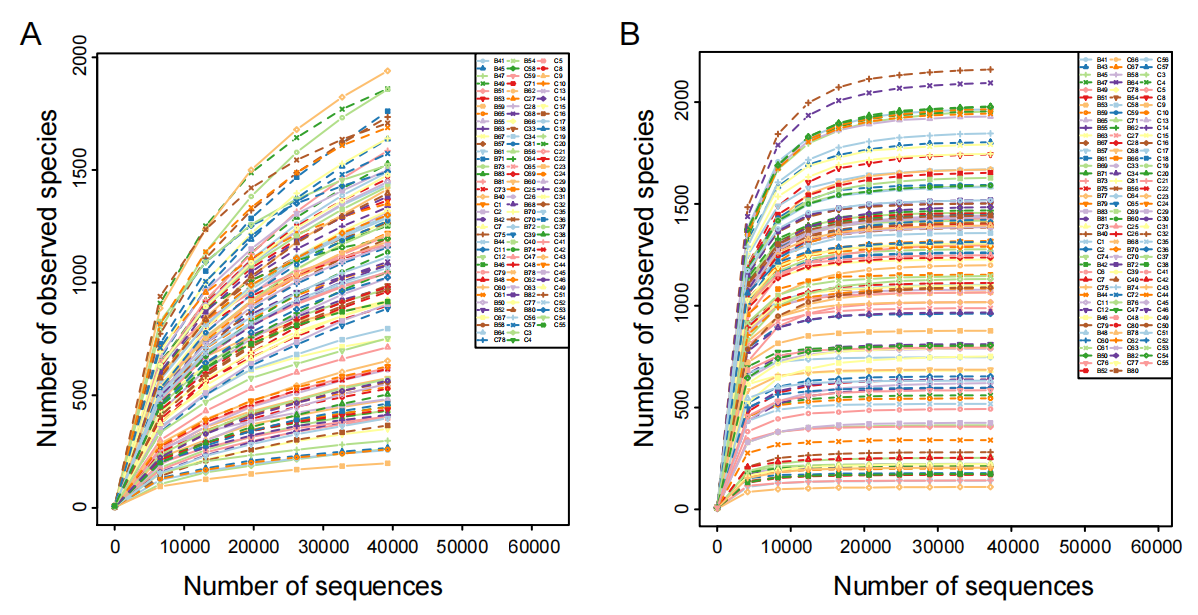

Supplement: Supplemental Information 1 — The number of fungal ITS and bacteria 16S rRNA sequences were normalized at 39, 262 and 37, 161, respectively. [file peerj-12-17225-s001.docx]
